# The dark side of ID8-Luc2: pitfalls for luciferase tagged murine models for ovarian cancer

**DOI:** 10.1186/s40425-015-0102-0

**Published:** 2015-12-15

**Authors:** Thaïs Baert, Tina Verschuere, Anaïs Van Hoylandt, Rik Gijsbers, Ignace Vergote, An Coosemans

**Affiliations:** Department of Gynaecology and Obstetrics, UZ Leuven, Leuven, Belgium; Department of Oncology, Laboratory of Gynaecologic Oncology, KU Leuven, Leuven Cancer Institute, Leuven, Belgium; Department of Neuroscience, Laboratory of Experimental Neurosurgery, KU Leuven, Leuven, Belgium; Department of Pharmaceutical and Pharmacological Sciences, Laboratory of Molecular Virology and Gene Therapy and Leuven Viral Vector Core, KU Leuven, Leuven, Belgium

**Keywords:** Ovarian cancer, ID8, Bioluminescence imaging, Mouse model, Ascites

## Abstract

Reliable mouse models are key in the discovery and development of novel anticancer treatments. Non-invasive monitoring techniques such as bioluminescence imaging (BLI) are useful tools to determine tumor engraftment and evaluate tumor growth. However, the development of ascites in ovarian cancer mouse models leads to possible difficulties. Ascites can interfere with the set-up of correct end points and can interfere with the evaluation of tumor volume using BLI. We provide optimized euthanasia criteria and in vivo data underlining the pitfalls of BLI.

## Background

With great interest, we read the article of Liao et al. entitled “Preservation of tumor-host immune interactions with luciferase-tagged imaging in a murine model of ovarian cancer” [1] in the Journal for ImmunoTherapy of Cancer. Adequate mouse models are paramount for translational cancer research. With the development of immunotherapy in the field of anticancer treatment, we should turn to immune competent syngeneic models such as the ID8-Luc2 model described by Liao et al.

Estimating and monitoring tumor load in ovarian cancer is challenging. With innumerable peritoneal implants that are formed, we rely in a clinical setting on CT (computed tomography) or MRI (magnetic resonance imaging) to evaluate tumor growth and disease progression, based on the RECIST 1.1 criteria [2]. The RECIST criteria use target lesions as surrogate measure for tumor load, as total tumor load cannot be quantified in a clinical setting. In animal models, bioluminescence imaging (BLI) is a well-established technique that allows non-invasive quantification of tumor load [3]. Typically, stably integrating retroviral vectors are used to generate stable luciferase expressing reporter lines, that are applied to the respective animal models to monitor tumor growth: cells that express the firefly reporter enzyme generate a photon flux (light) when luciferin (the luciferase substrate) is oxidized in the presence of ATP. As a consequence, only live tumor cells that express the enzyme can be monitored by detecting the emitted photons, rendering BLI an excellent and sensitive tool to examine tumor growth in mouse models, as was demonstrated nicely by the authors.

**Fig. 1:**
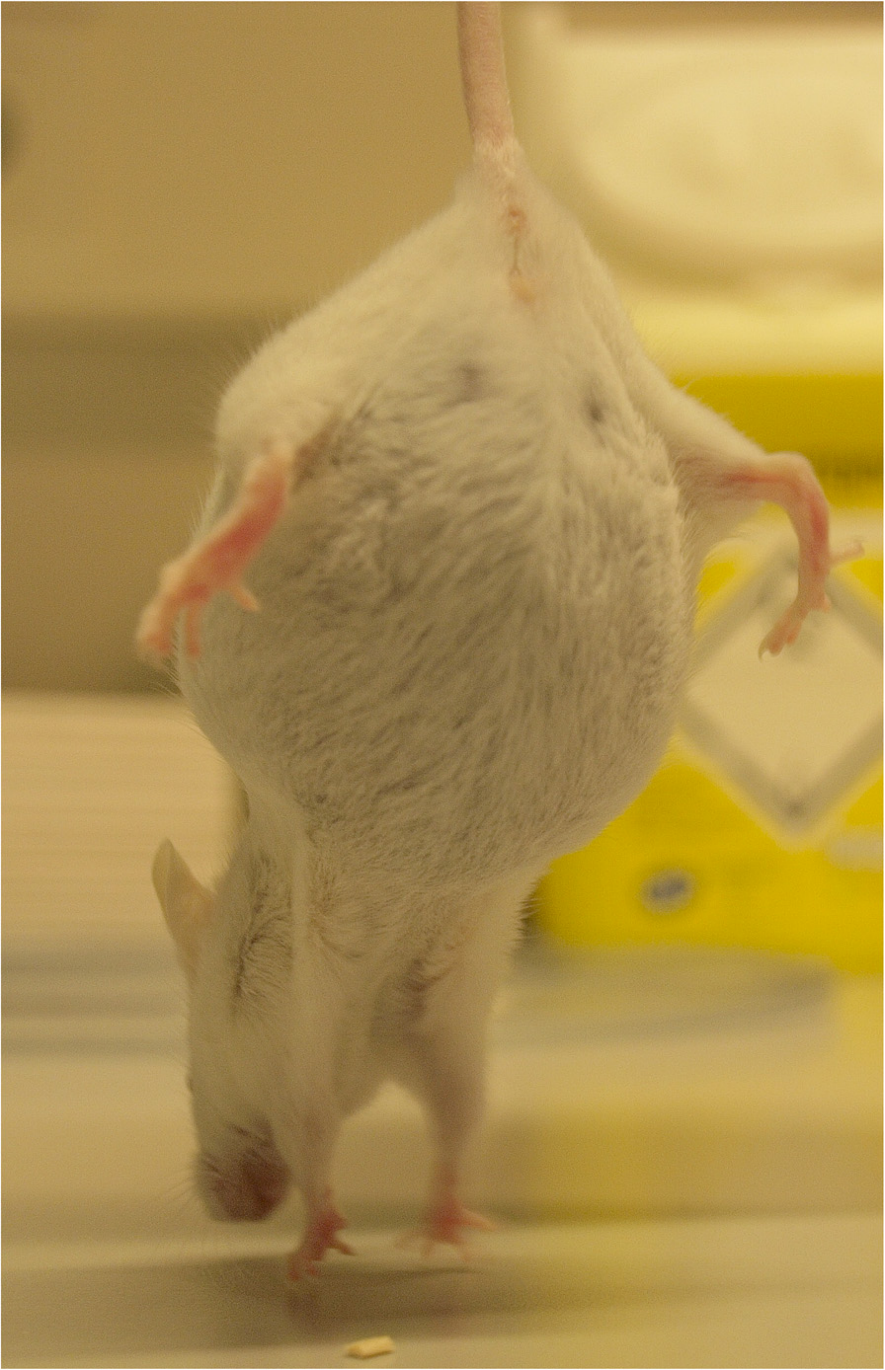
An example of a C57BL/6 J-Tyr^c-2J^/J mouse with ascites. Mouse inoculated with 10 × 10^6^ ID8-fLuc cells. Weight 32 g with appreciable ascites

**Fig. 2:**
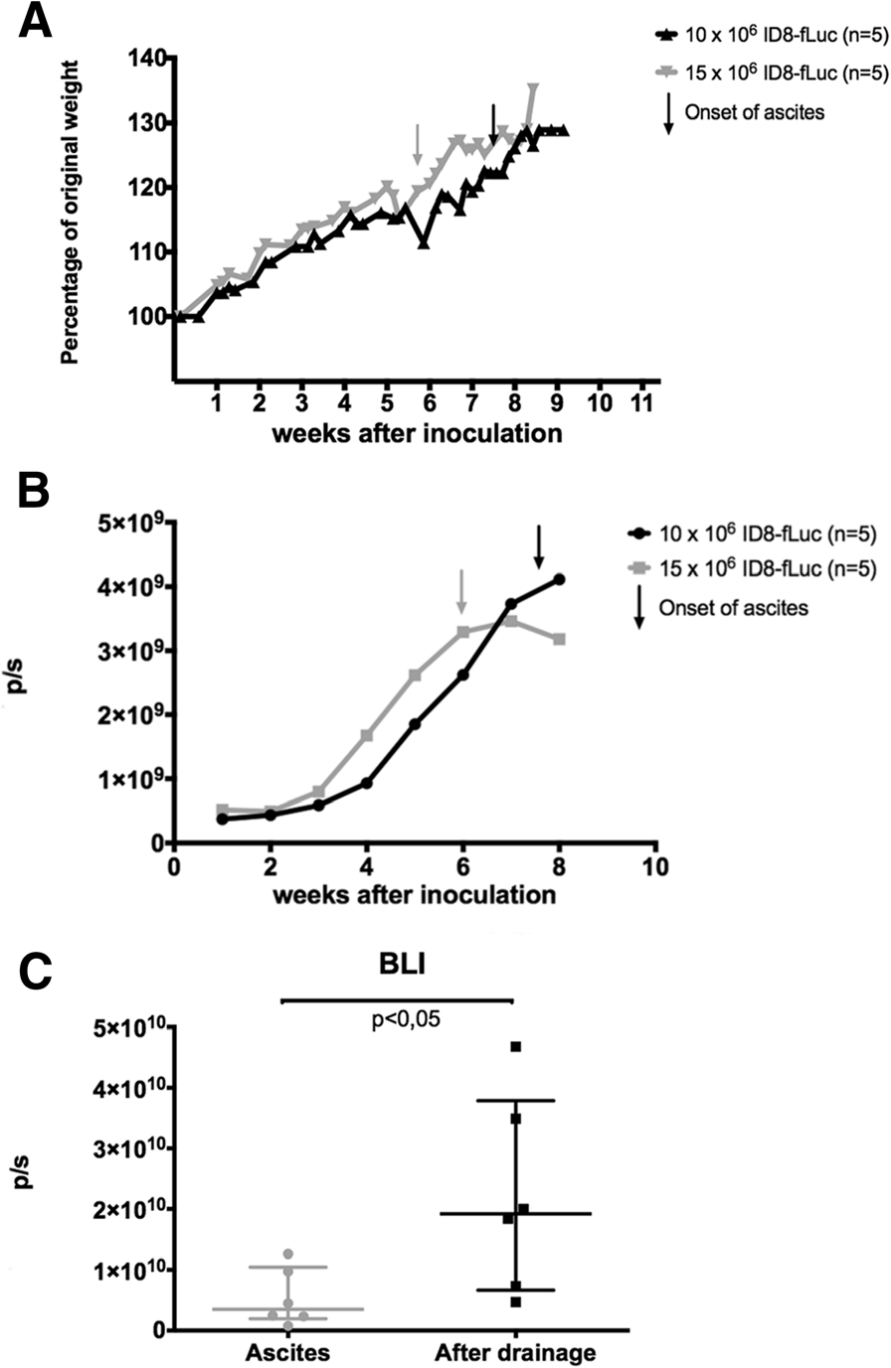
Weight curves and BLI signal of mice inoculated intraperitoneal with ID8-fLuc cells. **a** Weight curves of mice inoculated intraperitoneal with either 10 × 10^6^ or 15 × 10^6^ ID8-fLuc cells. Relative weight to weight at inoculation is used. When inoculating 15 × 10^6^ ID8-fLuc cells, ascites is clinically appreciable 6 weeks after inoculation, compared to 8 weeks after inoculation in the 10 × 10^6^ ID8-fLuc cells group. **b** BLI-signal of the animals described in 2A, weekly measurements in photons per second (p/s). Using pieceweise multilevel model we can show a statistical difference between the two groups up to week 6, when ascites arises in the 15 × 10^6^ ID8-fLuc group. **c** BLI results of mice with important ascites scanned without intervention or after drainage of ascites. We see a clear increase in the BLI signal after drainage of ascites. This is statistically significant using paired *t*-test. These results show that the presence of ascites decreases the BLI signal

Using BLI, we could even demonstrate established tumor growth after as little as one week after inoculation (minimum 10 x 10^6^ ID8-fLuc cells), long before macroscopic tumor or weight increase is detected. For further reference we will refer to the experiments performed by our research group with Firefly luciferase (luc1) transduced ID8 cell line as ID8-fLuc in contrast to ID8-Luc2 described by Liao et al. However, according to us, the technique also comes with significant shortcomings: is the photon flux a reliable measure for tumor volume after the onset of ascites (Fig. 1)? Luciferase activity is proportional to the number of cells that express the reporter, as long as the substrate luciferin is in abundance, and as long as ATP is available. For BLI we administer 126 mg/kg of luciferin in a concentration of 15 mg/L intraperitoneal (*ip*) to the mice [4]. However, in a mouse with ascites, the luciferin will be diluted in up to 15 mL of ascites, which consequently results in suboptimal substrate concentrations and accompanying photon flux and thus an underestimation of tumor load. When we inoculated mice with 15 × 10^6^ ID8-fLuc cells *ip* the mice developed clinically appreciable ascites 6 weeks after inoculation, in contrast to the group inoculated with 10 × 10^6^ ID8-fLuc cells, which developed ascites 8 weeks after tumor engraftment (Fig. 2a). Using a pieceweise multilevel model, we were able to show a significant difference in the BLI curves of the 10 × 10^6^ compared with the 15 × 10^6^ group until the 6^th^ week. To underscore that this stagnation of the BLI signal 6 weeks after inoculation in the 15 × 10^6^ ID8-fLuc group is linked to the presence of ascites we scanned mice with ascites before and after drainage of ascites.

Figure 2c shows significant increase in BLI signal after drainage of ascites. Therefore, we conclude that the occurrence of ascites affects the BLI photon flux and hence these measurements do not recapitulate the tumor growth. Especially if these mice receive treatments that can affect the development of ascites this is of particular importance. Moreover, in large tumor volumes the photon flux is mostly also an underestimate of tumor load, due to internal necrosis or weak vascularization of the tumor bulk, resulting in diminished/lower luciferin concentrations into the central zone of the tumor [5].

**Fig. 3:**
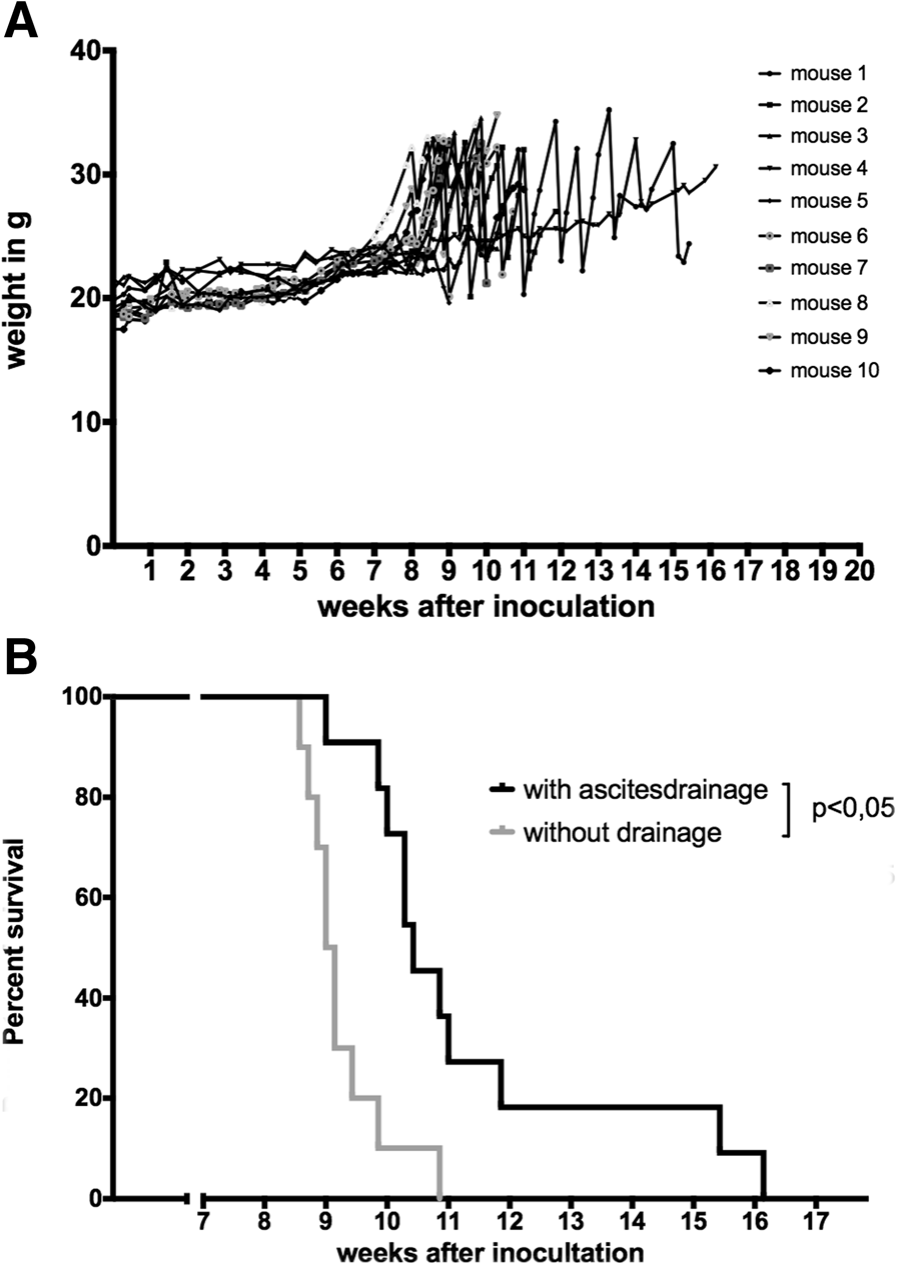
Ascites drainages in the ID8-fluc ovarian cancer mouse model. **a** Weight curves of mice inoculated intraperitoneal with 10 × 10^6^ ID8-fLuc cells. Absolute weights of individual animals are depicted. Results of two pooled experiments. **b** Kaplan-Meier curve showing survival of 10 mice inoculated with 10 × 106 ID8-fLuc as shown in a. The black curve depicts survival when draining the ascites and using our improved euthanasia criteria. The grey curve depicts survival when using the criteria described by Liao et al. Repetitive drainages of ascites lead to a significantly (*p* = 0,001) prolonged survival (Mantel-Cox)

Overall survival is the most important endpoint to determine the efficacy of an anticancer treatment. In animal models the strength of survival data depends on the criteria for euthanasia. In the study of Liao et al. clinical signs of disease or distress, interference of the tumor with normal bodily functions and development of ascites are used as end points. These criteria are vague and inter-observer variability is problematic. Moreover, the presence of ascites cannot be used as an end point. Ascites is a sign of widespread disease, but can be treated. Patients will in those cases undergo ascites drainage and/or initiation of chemotherapy. In an effort to mimic the clinical process as close as possible in our animal model set-up, we therefore decided to drain ascites in mice once they reached 32 g. Figure 3a shows an overview of the weight evolution in our experimental groups and the effect of draining ascites on the overall weight and survival. We could drain up to 12 mL (in average 8.81 mL ±2.97 mL) per treatment. Repetitive draining of ascites (up to 5 times) resulted in a significantly improved median survival from 63.5 to 73 days (Fig. 3b). We therefore conclude that ascites is not a good criterion for euthanasia to determine overall survival. As an alternative, we propose in Table 1 an overview of the euthanasia criteria we apply in our ID8-Fluc mouse model for ovarian cancer. These improved criteria provide more reproducible guidelines for the euthanasia of animals with ovarian cancer, without renouncing clinical relevance.

**Table 1:** Improved euthanasia criteria

Criteria of euthanasia	Liao et al.	Improved criteria
Weight	Cachexia	Loss of 2 g in 48 h
		Loss of 3 g in 7 days
Clinical deterioration	Increased respiratory frequency	Increased respiratory frequency
	Anorexia	Hunched back with tremor
	Ascites	/
	/	No spontaneous movement when nudged

Weight and clinical status should be evaluated once every 48 h. The improved criteria allow for more objective criteria for euthanasia of animals

**Fig. 4:**
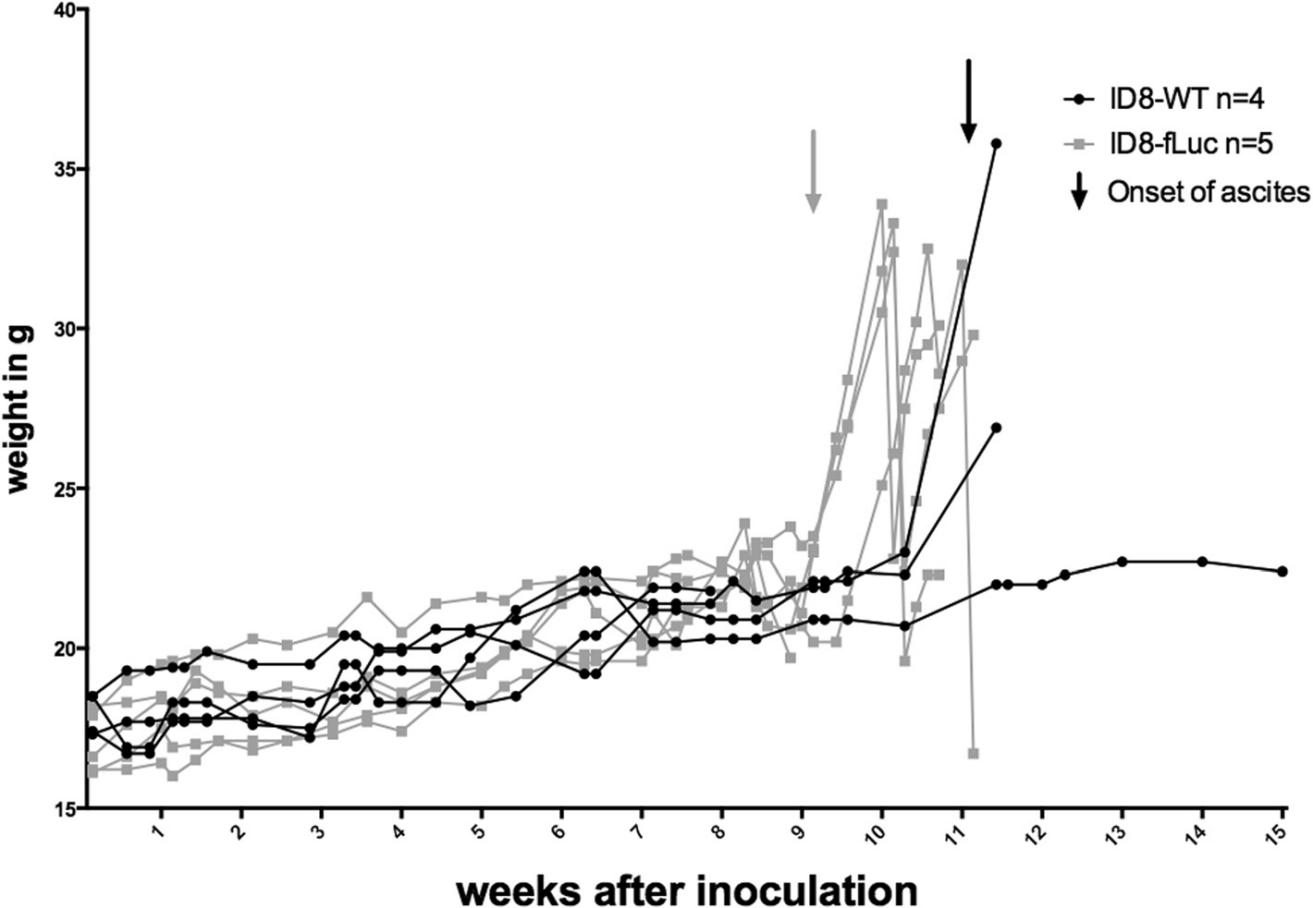
Comparison between the wild type (WT) and the fluc-transduced ID8 ovarian cancer mouse model. Weight curves of mice inoculated intraperitoneal with either 5 × 10^6^ ID8-WT or 5 × 10^6^ ID8-fLuc cells. Development of ascites occurs later in the group inoculated with ID8-WT cells. The differences in these weight curves are however non-significant

The authors spent a lot of effort in studying the changes in the humoral and cellular immunity due to the Luciferase insert. This is highly valuable information, especially for the use of the model in immunotherapy research. It is reassuring to see that although serum Luciferase IgG increases, it does not seem to have a detrimental influence on tumor growth represented by the BLI-signal. After transfection these cells express not only firefly Luciferase, but also the resistance protein (e.g., Puromycin resistance protein) on which they are selected. This protein is also a possible source of immunogenicity of the tumor. However, we would like to point out that in our model we noticed an increase in the in vivo tumor growth after stable lentiviral transduction and selection compared to the parental wild type ID8 (ID8-WT) model. As depicted in Fig. 4, onset of weight gain due to ascites occurs around day 65 after inoculation with 5 × 10^6^ ID8-fLuc cells, whereas this in only the case in 25 % of mice injected with ID8-WT (*n* = 1 out of 4 mice) at day 80. This suggests that there is no improved immune control of tumor growth in the transfected cell line, on the contrary, if anything the ID8-fLuc cell line grows faster in vivo when compared to the parental cell line.

In the article of Liao et al. FACS analysis was performed on ascites, spleen and tumor of ID8-WT inoculated mice compared to ID8-Luc2 inoculated mice at 12 to 15 weeks after inoculation to evaluate the tumor microenvironment. These are late stage mice in which immunosuppression has completely taken over from immune control. These results prove that in late stage animals there is no difference in the microenvironment due to the expression of luc2, but do not allow us to draw conclusions concerning early disease, when there is still an equilibrium between immune control and immune escape. Furthermore to determine the change in in vivo tumor growth due to the Luciferase insert, it would be more relevant to flank the firefly Luciferase cDNA with loxP sites, allowing Cre mediated excision of the cassette once the stable cell line is established, providing a perfect control. When comparing the cell line with and without the addition of Cre recombinase, the only difference is the presence firefly Luciferase and not the selection pressure that has been put on the cells during the transfection and selection process.

## Conclusion

In conclusion, the use of codon-optimized firefly luciferase Luc2 expressed in ID8 cells as described by Liao et al. is suitable for the evaluation of tumor load in early stage disease. Care should be taken in the interpretation of BLI results once ascites occurs. When using overall survival as an outcome measure in this model we recommend repeated ascites drainages to avoid underestimating survival. The use of orthotopic, immune competent models for ovarian cancer should be encouraged, as they are an adequate representation of the clinical setting of ovarian cancer patients.

## Abbreviations

BLI: Bioluminescence imaging
ID8-Luc2: Luc2 transfected cell line used by Liao et al.
ID8-fLuc: Luc1 transfected cell line used by our research group
ID8-WT: ID8 wild type, before transfection
p/s: Photons per second
CT: Computer tomography
MR: Magnetic resonance imaging
ip: Intraperitoneal
RECIST: Response evaluation criteria in solid tumors
